# The genome sequence of the willow leaf beetle, *Lochmaea capreae *Linnaeus, 1758

**DOI:** 10.12688/wellcomeopenres.22424.2

**Published:** 2024-10-17

**Authors:** Maxwell V. L. Barclay, Danaë Vassiliades, Will Bayfield-Farrell, Joana Cristóvão, Keita Matsumoto, Michael Geiser, Natural History Museum Genome Acquisition Lab, Darwin Tree of Life Barcoding collective, Wellcome Sanger Institute Tree of Life Management, Samples and Laboratory team, Wellcome Sanger Institute Scientific Operations: Sequencing Operations, Wellcome Sanger Institute Tree of Life Core Informatics team, Tree of Life Core Informatics collective, Darwin Tree of Life Consortium

**Affiliations:** 1 Natural History Museum, London, England, UK

**Keywords:** Lochmaea capreae, willow leaf beetle, genome sequence, chromosomal, Coleoptera

## Abstract

We present a genome assembly from an individual female *Lochmaea capreae* (the willow leaf beetle; Arthropoda; Insecta; Coleoptera; Chrysomelidae). The genome sequence is 534.7 megabases in span. Most of the assembly is scaffolded into 17 chromosomal pseudomolecules. The mitochondrial genome has also been assembled and is 18.85 kilobases in length. Gene annotation of this assembly on Ensembl identified 12,254 protein coding genes.

## Species taxonomy

Eukaryota; Opisthokonta; Metazoa; Eumetazoa; Bilateria; Protostomia; Ecdysozoa; Panarthropoda; Arthropoda; Mandibulata; Pancrustacea; Hexapoda; Insecta; Dicondylia; Pterygota; Neoptera; Endopterygota; Coleoptera; Polyphaga; Cucujiformia; Chrysomeloidea; Chrysomelidae; Galerucinae; Galerucini; *Lochmaea*; *Lochmaea capreae* Linnaeus, 1758 (NCBI:txid227480).

## Background

This is the second Darwin Tree of Life data note concerning the genus *Lochmaea* Weise, 1883 after *L. crataegi* was treated earlier this year ( Crowley *et al*., 2024). *Lochmaea* is a Palaearctic genus with 15 valid species ( Lee, 2019), of which three are found in the UK ( Duff, 2018). The spelling “ *caprea*” for the species name is adopted here in accordance with Beenen (2010) and Duff (2018), who consider the widely used spelling “ *capreae*” to be an unjustified emendation of Linnaeus’ name.

The three British species of the genus are all widespread within the Palaearctic region. *L. caprea* ranges from northern Spain until Korea and Japan ( Beenen, 2010). The eastern Palaearctic populations are sometimes classified as a separate subspecies, *L. caprea cribrata* Solsky, 1872 ( Warchałowski, 2010). A related species, *L. scutellata* (Chevrolat, 1840) occurs on the Iberian Peninsula. For most of its European range, including Britain and Ireland, *L. caprea* overlaps in range with another close relative, the “Heather Leaf Beetle” *L. suturalis* (C.G. Thomson, 1866), with which it can sometimes be confused.

*L. caprea* is a medium-sized (4–6 mm) brownish leaf beetle with black ventral side, head, femora, tarsi, palpi and antennae (except some of the basal antennomeres). Live beetles often have paler yellowish-brown elytra than dry museum specimens. The pale elytral colour is an easy, albeit not entirely reliable way to distinguish it from *L. suturalis*. In *L. suturalis*, the elytral suture is almost always dark brown or black. Particularly in the North of the British Isles, melanistic forms with almost completely dark brownish dorsal side tend to be common. There are occasional colour variations of *L. suturalis* without the dark elytral suture, which can be easily confused with *L. caprea*. In those cases, *L. suturalis* has characteristically yellow antennal insertions on the head, while *L. caprea* always has a completely black head. Melanistic individuals of *L. suturalis* can also have black antennal insertions but are otherwise distinguishable from *L. caprea* by the elytral colour ( Mohr, 1966). Another diagnostic character for *L. caprea* is the isodametric micro-reticulation of the pronotum, visible at higher magnifications ( Duff, 2016). The male genitalia of both species are rather distinct in their shape, so this can be used as a reliable character if unusual colour forms are suspected ( Duff, 2016; Warchałowski, 2010). Furthermore, *L. suturalis* is strictly associated with heather ( *Calluna vulgaris*), while *L. caprea* has different host plants.

As its common name “Willow leaf beetle” suggests, *L. caprea* is most often found associated with willows, most frequently *Salix caprea* (Goat Willow). It has, however, also been recorded to feed on *Betula* (birch), *Alnus incana* (grey alder), *Populus* (poplar) and even *Rhododendron tomentosum* ( Rheinheimer & Hassler, 2018). Both adults and larvae feed externally on leaves of their host plant. Following the distribution of its hosts, *L. caprea* is most commonly seen in wet habitats, often along bodies of water, but can be found in a wide variety of habitats ( Rheinheimer & Hassler, 2018).

*Lochmaea caprea* is generally univoltine. Adult activity starts in April, after the winter is spent in diapause. May and June see the highest abundance of adults, which will then deposit their eggs on their host plants and die off in July. Larval development and pupation generally take place between May and August, with the new generation of adults hatching in late summer ( Rheinheimer & Hassler, 2018). Larval stages can be identified using Steinhausen (1994). The eulophid wasp *Chrysocharis nitetis* (Walker, 1839) is known to parasitise larvae of *L. caprea*, along with other insect hosts ( Rheinheimer & Hassler, 2018).

*L. caprea* is a common and widely distributed species on the British Isles, though the density of records is higher in England and Wales than it is in Scotland and Ireland ( Cox, 2007). It is listed as “Least Concern” in Hubble (2014).

The genome of the willow leaf beetle, *Lochmaea capreae*, was sequenced as part of the Darwin Tree of Life Project, a collaborative effort to sequence all named eukaryotic species in the Atlantic Archipelago of Britain and Ireland. Here we present a chromosomally complete genome sequence for *Lochmaea capreae*, based on one female specimen from Bookham Commons England.

## Genome sequence report

**Figure 1.**
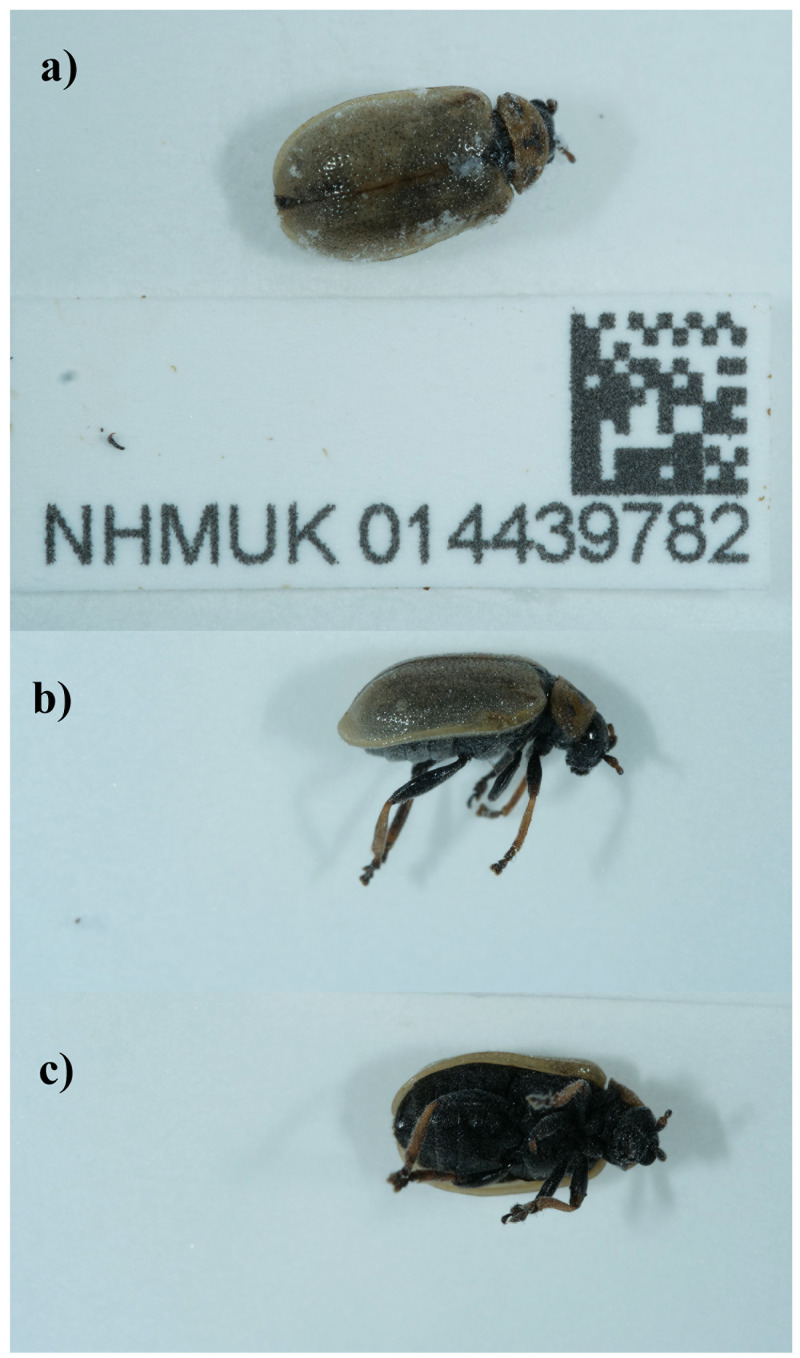
Photograph of the *Lochmaea capreae* (icLocCapr1) specimen used for genome sequencing.

The genome was sequenced from one female *Lochmaea capreae* ( Figure 1) collected from Bookham Commons, England, UK (51.29, –0.39). A total of 48-fold coverage in Pacific Biosciences single-molecule HiFi long reads was generated. Primary assembly contigs were scaffolded with chromosome conformation Hi-C data. Manual assembly curation corrected 29 missing joins or mis-joins and removed 6 haplotypic duplications, reducing the assembly length by 0.65% and the scaffold number by 27.27%, and increasing the scaffold N50 by 2.07%.

**Figure 2.**
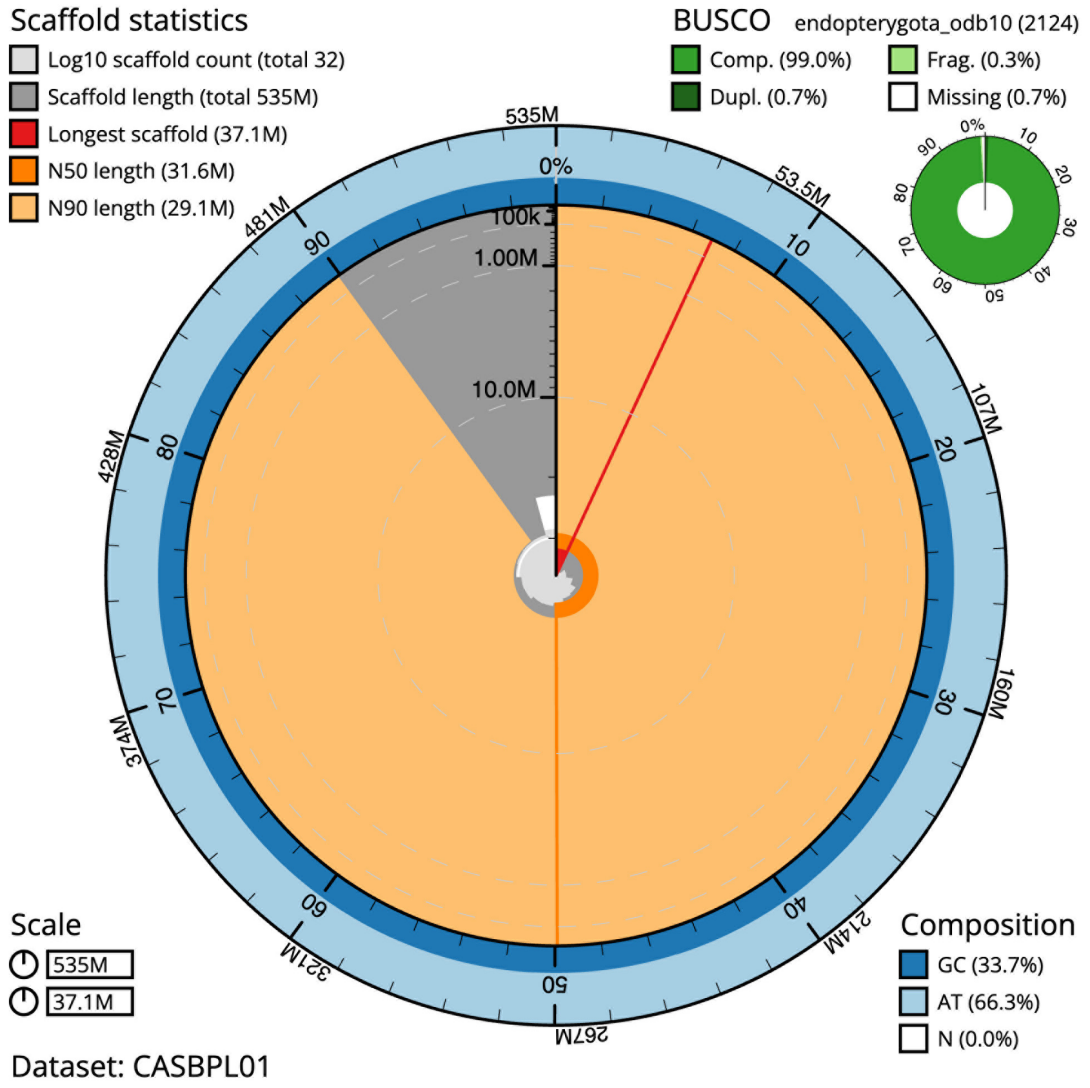
Genome assembly of *Lochmaea capreae*, icLocCapr1.1: metrics. The BlobToolKit Snailplot shows N50 metrics and BUSCO gene completeness. The main plot is divided into 1,000 size-ordered bins around the circumference with each bin representing 0.1% of the 534,692,516 bp assembly. The distribution of scaffold lengths is shown in dark grey with the plot radius scaled to the longest scaffold present in the assembly (37,061,687 bp, shown in red). Orange and pale-orange arcs show the N50 and N90 scaffold lengths (31,551,978 and 29,064,511 bp), respectively. The pale grey spiral shows the cumulative scaffold count on a log scale with white scale lines showing successive orders of magnitude. The blue and pale-blue area around the outside of the plot shows the distribution of GC, AT and N percentages in the same bins as the inner plot. A summary of complete, fragmented, duplicated and missing BUSCO genes in the endopterygota_odb10 set is shown in the top right. An interactive version of this figure is available at https://blobtoolkit.genomehubs.org/view/CASBPL01/dataset/CASBPL01/snail.

**Figure 3.**
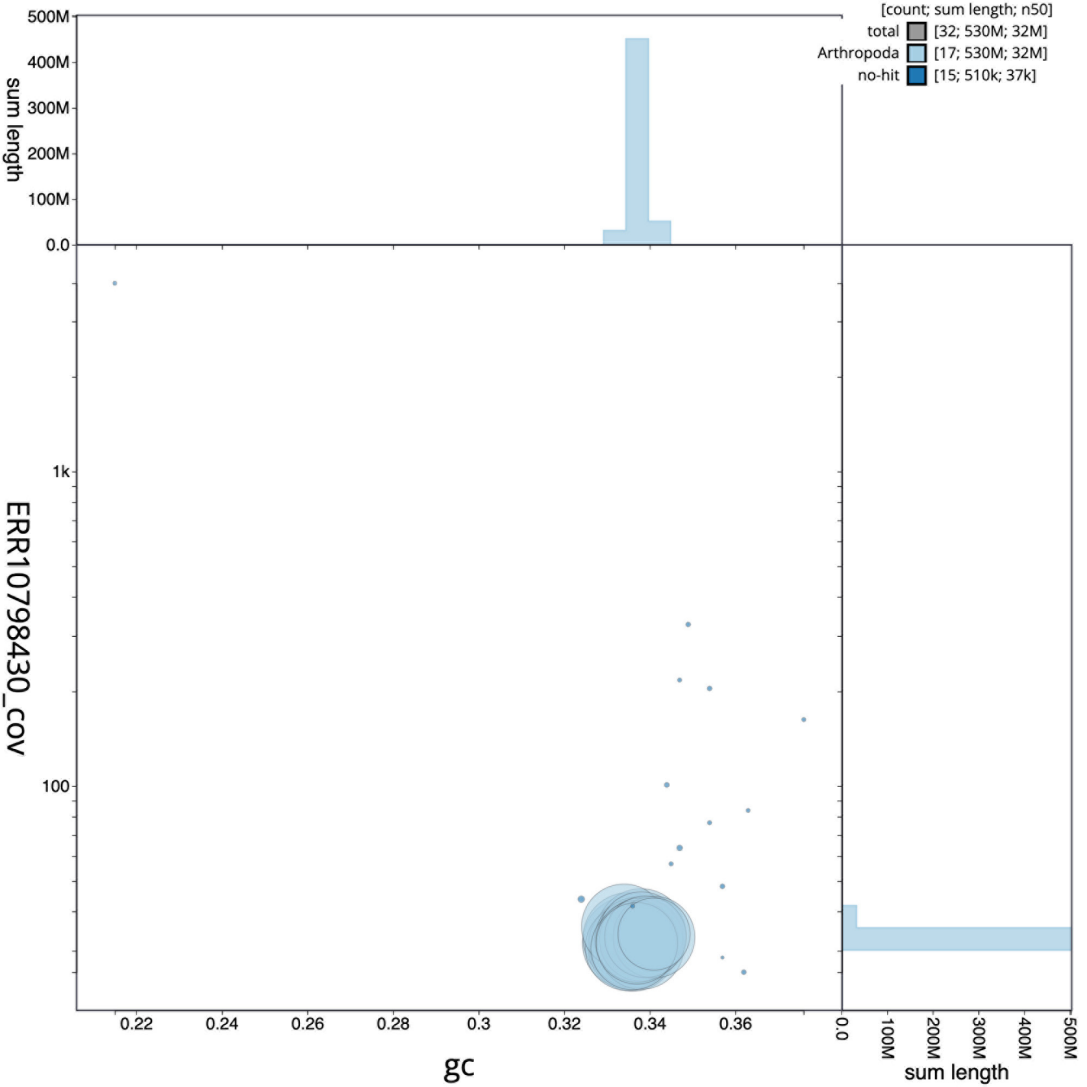
Genome assembly of *Lochmaea capreae*, icLocCapr1.1: BlobToolKit GC-coverage plot. Scaffolds are coloured by phylum. Circles are sized in proportion to scaffold length. Histograms show the distribution of scaffold length sum along each axis. An interactive version of this figure is available at https://blobtoolkit.genomehubs.org/view/CASBPL01/dataset/CASBPL01/blob.

**Figure 4.**
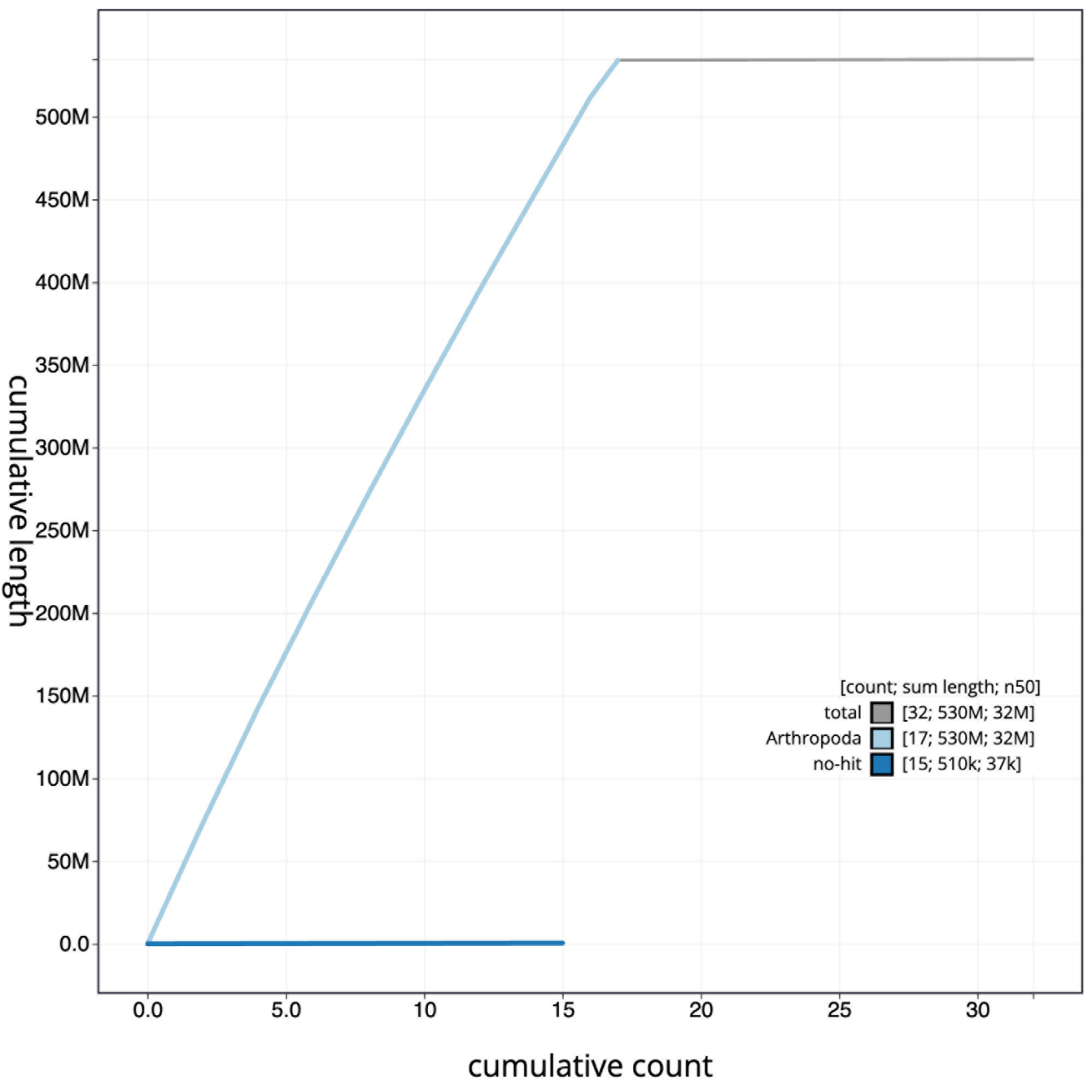
Genome assembly of *Lochmaea capreae*, icLocCapr1.1: BlobToolKit cumulative sequence plot. The grey line shows cumulative length for all scaffolds. Coloured lines show cumulative lengths of scaffolds assigned to each phylum using the buscogenes taxrule. An interactive version of this figure is available at https://blobtoolkit.genomehubs.org/view/CASBPL01/dataset/CASBPL01/cumulative.

**Figure 5.**
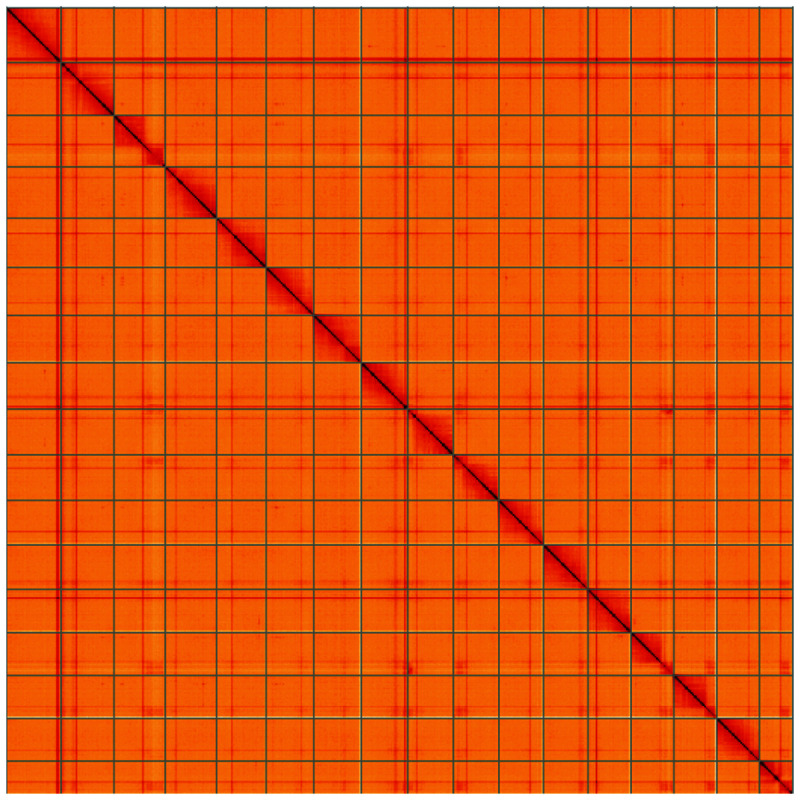
Genome assembly of *Lochmaea capreae*, icLocCapr1.1: Hi-C contact map of the icLocCapr1.1 assembly, visualised using HiGlass. Chromosomes are shown in order of size from left to right and top to bottom. An interactive version of this figure may be viewed at https://genome-note-higlass.tol.sanger.ac.uk/l/?d=UAgatg54QvqIWQyLAY1m7w.

The final assembly has a total length of 534.7 Mb in 31 sequence scaffolds with a scaffold N50 of 31.6 Mb ( Table 1). The snail plot in Figure 2 provides a summary of the assembly statistics, while the distribution of assembly scaffolds on GC proportion and coverage is shown in Figure 3. The cumulative assembly plot in Figure 4 shows curves for subsets of scaffolds assigned to different phyla. Most (99.9%) of the assembly sequence was assigned to 17 chromosomal-level scaffolds. Chromosome-scale scaffolds confirmed by the Hi-C data are named in order of size ( Figure 5; Table 2). The specimen is homogametic and thus female, but the X chromosome could not be confirmed. Chromosomes 8 and 16 both align to the annotated X chromosome in *Lochmaea crataegi* (GCA_947563755.1) ( Crowley *et al.*, 2024). While not fully phased, the assembly deposited is of one haplotype. Contigs corresponding to the second haplotype have also been deposited. The mitochondrial genome was also assembled and can be found as a contig within the multifasta file of the genome submission.

**Table 1.** Genome data for *Lochmaea capreae*, icLocCapr1.1.

Project accession data		
Assembly identifier	icLocCapr1.1	
Species	*Lochmaea capreae*	
Specimen	icLocCapr1	
NCBI taxonomy ID	227480	
BioProject	PRJEB59073	
BioSample ID	SAMEA14448388	
Isolate information	icLocCapr1	
Assembly metrics *		*Benchmark*
Consensus quality (QV)	68.5	*≥ 50*
*k*-mer completeness	100.0%	*≥ 95%*
BUSCO **	C:99.0%[S:98.3%,D:0.7%], F:0.3%,M:0.7%,n:2,124	*C ≥ 95%*
Percentage of assembly mapped to chromosomes	99.9%	*≥ 95%*
Sex chromosomes	None	*localised homologous* *pairs*
Organelles	Mitochondrial genome: 18.85 kb	*complete single alleles*
Raw data accessions		
PacificBiosciences SEQUEL II	ERR10798430	
Hi-C Illumina	ERR10802451	
PolyA RNA-Seq Illumina	ERR11242519	
Genome assembly		
Assembly accession	GCA_949126875.1	
*Accession of alternate haplotype*	GCA_949126855.1	
Span (Mb)	534.7	
Number of contigs	105	
Contig N50 length (Mb)	9.5	
Number of scaffolds	31	
Scaffold N50 length (Mb)	31.6	
Longest scaffold (Mb)	37.06	
Genome annotation		
Number of protein-coding genes	12,254	
Number of non-coding genes	1,518	
Number of gene transcripts	20,438	

* Assembly metric benchmarks are adapted from column VGP-2020 of “Table 1: Proposed standards and metrics for defining genome assembly quality” from ( Rhie *et al.*, 2021). ** BUSCO scores based on the endopterygota_odb10 BUSCO set using version 5.3.2. C = complete [S = single copy, D = duplicated], F = fragmented, M = missing, n = number of orthologues in comparison. A full set of BUSCO scores is available at https://blobtoolkit.genomehubs.org/view/CASBPL01/dataset/CASBPL01/busco.

**Table 2.** Chromosomal pseudomolecules in the genome assembly of *Lochmaea capreae*, icLocCapr1.

INSDC accession	Chromosome	Length (Mb)	GC%
OX421395.1	1	37.06	33.5
OX421396.1	2	35.97	33.5
OX421397.1	3	34.9	34.0
OX421398.1	4	34.88	33.5
OX421399.1	5	33.57	33.5
OX421400.1	6	32.45	33.5
OX421401.1	7	32.2	34.0
OX421402.1	8	31.55	33.5
OX421403.1	9	30.99	33.5
OX421404.1	10	30.91	34.0
OX421405.1	11	30.32	33.5
OX421406.1	12	30.23	34.0
OX421407.1	13	29.3	33.5
OX421408.1	14	29.16	34.0
OX421409.1	15	29.06	34.0
OX421410.1	16	29.0	33.5
OX421411.1	17	22.62	34.0
OX421412.1	MT	0.02	21.5

The estimated Quality Value (QV) of the final assembly is 68.5 with *k*-mer completeness of 100.0%, and the assembly has a BUSCO v5.3.2 completeness of 99.0% (single = 98.3%, duplicated = 0.7%), using the endopterygota_odb10 reference set ( *n* = 2,124).

Metadata for specimens, barcode results, spectra estimates, sequencing runs, contaminants and pre-curation assembly statistics are given at https://links.tol.sanger.ac.uk/species/227480.

## Genome annotation report

The *Lochmaea capreae* genome assembly (GCA_949126875.1) was annotated using the Ensembl rapid annotation pipeline ( Table 1; https://rapid.ensembl.org/Lochmaea_capreae_GCA_949126875.1/Info/Index). The resulting annotation includes 20,438 transcribed mRNAs from 12,254 protein-coding and 1,518 non-coding genes.

## Methods

### Sample acquisition and nucleic acid extraction

A female *Lochmaea capreae* (specimen ID NHMUK014439782, ToLID icLocCapr1) was collected from Bookham Commons, England, UK (latitude 51.29, longitude –0.39) on 2021-09-19. The specimen was collected by Maxwell Barclay, Danaë Vassiliades, Will Bayfield Farrell, Joana Cristovao and Keita Matsumoto (all Natural History Museum) and identified by Michael Geiser (Natural History Museum), and then preserved by dry freezing at –80 °C.

Protocols developed by the Wellcome Sanger Institute (WSI) Tree of Life core laboratory have been deposited on protocols.io ( Denton *et al.*, 2023b). The workflow for high molecular weight (HMW) DNA extraction at the WSI includes a sequence of core procedures: sample preparation; sample homogenisation, DNA extraction, fragmentation, and clean-up. In sample preparation, the icLocCapr1 sample was weighed and dissected on dry ice ( Jay *et al.*, 2023). Tissue from the abdomen was homogenised using a PowerMasher II tissue disruptor ( Denton *et al.*, 2023a).

HMW DNA was extracted using the Automated MagAttract v1 protocol ( Oatley *et al.*, 2023a). HMW DNA was sheared into an average fragment size of 12–20 kb in a Megaruptor 3 system with speed setting 30 ( Todorovic *et al.*, 2023). Sheared DNA was purified by solid-phase reversible immobilisation ( Oatley *et al.*, 2023b): using AMPure PB beads to eliminate shorter fragments and concentrate the DNA. The concentration of the sheared and purified DNA was assessed using a Nanodrop spectrophotometer and Qubit Fluorometer and Qubit dsDNA High Sensitivity Assay kit. Fragment size distribution was evaluated by running the sample on the FemtoPulse system.

RNA was extracted from leg tissue of icLocCapr1 in the Tree of Life Laboratory at the WSI using the RNA Extraction: Automated MagMax™ *mir*Vana protocol ( do Amaral *et al.*, 2023). The RNA concentration was assessed using a Nanodrop spectrophotometer and a Qubit Fluorometer using the Qubit RNA Broad-Range Assay kit. Analysis of the integrity of the RNA was done using the Agilent RNA 6000 Pico Kit and Eukaryotic Total RNA assay.

### Sequencing

Pacific Biosciences HiFi circular consensus DNA sequencing libraries were constructed according to the manufacturers’ instructions. Poly(A) RNA-Seq libraries were constructed using the NEB Ultra II RNA Library Prep kit. DNA and RNA sequencing was performed by the Scientific Operations core at the WSI on Pacific Biosciences SEQUEL II (HiFi) and Illumina NovaSeq 6000 (RNA-Seq) instruments. Hi-C data were also generated from head and thorax tissue of icLocCapr1 using the Arima2 kit and sequenced on the Illumina NovaSeq 6000 instrument.

### Genome assembly, curation and evaluation

Assembly was carried out with Hifiasm ( Cheng *et al.*, 2021) and haplotypic duplication was identified and removed with purge_dups ( Guan *et al.*, 2020). The assembly was then scaffolded with Hi-C data ( Rao *et al.*, 2014) using YaHS ( Zhou *et al.*, 2023). The assembly was checked for contamination and corrected as described previously ( Howe *et al.*, 2021). Manual curation was performed using HiGlass ( Kerpedjiev *et al.*, 2018) and PretextView ( Harry, 2022). The mitochondrial genome was assembled using MitoHiFi ( Uliano-Silva *et al.*, 2023), which runs MitoFinder ( Allio *et al.*, 2020) or MITOS (Bernt *et al.*, 2013) and uses these annotations to select the final mitochondrial contig and to ensure the general quality of the sequence.

A Hi-C map for the final assembly was produced using bwa-mem2 ( Vasimuddin *et al.*, 2019) in the Cooler file format ( Abdennur & Mirny, 2020). To assess the assembly metrics, the *k*-mer completeness and QV consensus quality values were calculated in Merqury ( Rhie *et al.*, 2020). This work was done using Nextflow ( Di Tommaso *et al.*, 2017) DSL2 pipelines “sanger-tol/readmapping” ( Surana *et al.*, 2023a) and “sanger-tol/genomenote” ( Surana *et al.*, 2023b). The genome was analysed within the BlobToolKit environment ( Challis *et al.*, 2020) and BUSCO scores ( Manni *et al.*, 2021; Simão *et al.*, 2015) were calculated.

Table 3 contains a list of relevant software tool versions and sources.

**Table 3.** Software tools: versions and sources.

Software tool	Version	Source
BlobToolKit	4.1.7	https://github.com/blobtoolkit/blobtoolkit
BUSCO	5.3.2	https://gitlab.com/ezlab/busco
Hifiasm	0.16.1-r375	https://github.com/chhylp123/hifiasm
HiGlass	1.11.6	https://github.com/higlass/higlass
Merqury	MerquryFK	https://github.com/thegenemyers/MERQURY.FK
MitoHiFi	2	https://github.com/marcelauliano/MitoHiFi
PretextView	0.2	https://github.com/wtsi-hpag/PretextView
purge_dups	1.2.3	https://github.com/dfguan/purge_dups
sanger-tol/genomenote	v1.0	https://github.com/sanger-tol/genomenote
sanger-tol/readmapping	1.1.0	https://github.com/sanger-tol/readmapping/tree/1.1.0
YaHS	1.2a	https://github.com/c-zhou/yahs

### Genome annotation

The Ensembl gene annotation system ( Aken *et al.*, 2016) was used to generate annotation for the *Lochmaea capreae* assembly (GCA_949126875.1). Annotation was created primarily through alignment of transcriptomic data to the genome, with gap filling via protein-to-genome alignments of a select set of proteins from UniProt ( UniProt Consortium, 2019).

### Wellcome Sanger Institute – Legal and Governance

The materials that have contributed to this genome note have been supplied by a Darwin Tree of Life Partner. The submission of materials by a Darwin Tree of Life Partner is subject to the **‘Darwin Tree of Life Project Sampling Code of Practice’**, which can be found in full on the Darwin Tree of Life website here. By agreeing with and signing up to the Sampling Code of Practice, the Darwin Tree of Life Partner agrees they will meet the legal and ethical requirements and standards set out within this document in respect of all samples acquired for, and supplied to, the Darwin Tree of Life Project. 

Further, the Wellcome Sanger Institute employs a process whereby due diligence is carried out proportionate to the nature of the materials themselves, and the circumstances under which they have been/are to be collected and provided for use. The purpose of this is to address and mitigate any potential legal and/or ethical implications of receipt and use of the materials as part of the research project, and to ensure that in doing so we align with best practice wherever possible. The overarching areas of consideration are:

•      Ethical review of provenance and sourcing of the material

•      Legality of collection, transfer and use (national and international) 

Each transfer of samples is further undertaken according to a Research Collaboration Agreement or Material Transfer Agreement entered into by the Darwin Tree of Life Partner, Genome Research Limited (operating as the Wellcome Sanger Institute), and in some circumstances other Darwin Tree of Life collaborators.

## Data Availability

European Nucleotide Archive: *Lochmaea capreae* (willow leaf beetle). Accession number PRJEB59073; https://identifiers.org/ena.embl/PRJEB59073 ( Wellcome Sanger Institute, 2023). The genome sequence is released openly for reuse. The *Lochmaea capreae* genome sequencing initiative is part of the Darwin Tree of Life (DToL) project. All raw sequence data and the assembly have been deposited in INSDC databases. Raw data and assembly accession identifiers are reported in Table 1.

## Author information

Members of the Natural History Museum Genome Acquisition Lab are listed here: https://doi.org/10.5281/zenodo.7139035.

Members of the Darwin Tree of Life Barcoding collective are listed here: https://doi.org/10.5281/zenodo.4893703.

Members of the Wellcome Sanger Institute Tree of Life Management, Samples and Laboratory team are listed here: https://doi.org/10.5281/zenodo.10066175.

Members of Wellcome Sanger Institute Scientific Operations: Sequencing Operations are listed here: https://doi.org/10.5281/zenodo.10043364.

Members of the Wellcome Sanger Institute Tree of Life Core Informatics team are listed here: https://doi.org/10.5281/zenodo.10066637.

Members of the Tree of Life Core Informatics collective are listed here: https://doi.org/10.5281/zenodo.5013541.

Members of the Darwin Tree of Life Consortium are listed here: https://doi.org/10.5281/zenodo.4783558.

## References

[ref-1] Abdennur N Mirny LA : Cooler: scalable storage for Hi-C data and other genomically labeled arrays. *Bioinformatics.* 2020;36(1):311–316. 10.1093/bioinformatics/btz540 31290943 PMC8205516

[ref-2] Aken BL Ayling S Barrell D : The ensembl gene annotation system. *Database (Oxford).* 2016;2016: baw093. 10.1093/database/baw093 27337980 PMC4919035

[ref-3] Allio R Schomaker-Bastos A Romiguier J : MitoFinder: efficient automated large-scale extraction of mitogenomic data in target enrichment phylogenomics. *Mol Ecol Resour.* 2020;20(4):892–905. 10.1111/1755-0998.13160 32243090 PMC7497042

[ref-31] Beenen R : Subfamily galerucinae latreille, 1802.In: Löbl, I. & Smetana, A. (eds.) *Catalogue of Palaearctic Coleoptera.*Chrysomeloidea. Stenstrup: Apollo Books,2010;6:924.

[ref-5] Challis R Richards E Rajan J : BlobToolKit – interactive quality assessment of genome assemblies. *G3 (Bethesda).* 2020;10(4):1361–1374. 10.1534/g3.119.400908 32071071 PMC7144090

[ref-6] Cheng H Concepcion GT Feng X : Haplotype-resolved *de novo* assembly using phased assembly graphs with hifiasm. *Nat Methods.* 2021;18(2):170–175. 10.1038/s41592-020-01056-5 33526886 PMC7961889

[ref-32] Cox ML : Atlas of the seed and leaf beetles of Britain.Newbery: Pisces Publications,2007;336. Reference Source

[ref-33] Crowley LM Telfer MG Escalona HE : The genome sequence of the hawthorn leaf beetle, *Lochmaea crataegi* (Forster, 1771) [version 1; peer review: 2 approved]. *Wellcome Open Res.* 2024;9:80. 10.12688/wellcomeopenres.20911.1 39246515 PMC11380073

[ref-7] Denton A Oatley G Cornwell C : Sanger Tree of Life sample homogenisation: PowerMash. *protocols.io.* 2023a. 10.17504/protocols.io.5qpvo3r19v4o/v1

[ref-8] Denton A Yatsenko H Jay J : Sanger Tree of Life wet laboratory protocol collection V.1. *protocols.io.* 2023b. 10.17504/protocols.io.8epv5xxy6g1b/v1

[ref-9] Di Tommaso P Chatzou M Floden EW : Nextflow enables reproducible computational workflows. *Nat Biotechnol.* 2017;35(4):316–319. 10.1038/nbt.3820 28398311

[ref-10] do Amaral RJV Bates A Denton A : Sanger Tree of Life RNA extraction: automated MagMax ^TM^ mirVana. *protocols.io.* 2023. 10.17504/protocols.io.6qpvr36n3vmk/v1

[ref-34] Duff AG : Beetles of Britain and Ireland.Cerambycidae to Curculionidae. West Runton: A.G. Duff publishing,2016;4:623. Reference Source

[ref-35] Duff AG : Checklist of beetles of the British Isles.3rd Edition. Iver: Pemberley Books,2018;248. Reference Source

[ref-11] Guan D McCarthy SA Wood J : Identifying and removing haplotypic duplication in primary genome assemblies. *Bioinformatics.* 2020;36(9):2896–2898. 10.1093/bioinformatics/btaa025 31971576 PMC7203741

[ref-12] Harry E : PretextView (Paired Read Texture Viewer): a desktop application for viewing pretext contact maps. 2022; [Accessed 19 October 2022]. Reference Source

[ref-13] Howe K Chow W Collins J : Significantly improving the quality of genome assemblies through curation. *GigaScience.* Oxford University Press,2021;10(1): giaa153. 10.1093/gigascience/giaa153 33420778 PMC7794651

[ref-36] Hubble DS : A review of the scarce and threatened beetles of Great Britain. The leaf beetles and their allies, Chrysomelidae, Megalopodidae and Orsodacnidae.Species Status No. 19. Natural England,2014;118. Reference Source

[ref-14] Jay J Yatsenko H Narváez-Gómez JP : Sanger Tree of Life sample preparation: triage and dissection. *protocols.io.* 2023. 10.17504/protocols.io.x54v9prmqg3e/v1

[ref-15] Kerpedjiev P Abdennur N Lekschas F : Higlass: web-based visual exploration and analysis of genome interaction maps. *Genome Biol.* 2018;19(1): 125. 10.1186/s13059-018-1486-1 30143029 PMC6109259

[ref-37] Lee CF : The genus *Lochmaea* Weise, 1883 in Taiwan: results of taxonomic expeditions by citizen scientists (Coleoptera, Chrysomelidae, Galerucinae). *ZooKeys.* 2019;856:75–100. 10.3897/zookeys.856.30838 31258368 PMC6591211

[ref-16] Manni M Berkeley MR Seppey M : BUSCO update: novel and streamlined workflows along with broader and deeper phylogenetic coverage for scoring of eukaryotic, prokaryotic, and viral genomes. *Mol Biol Evol.* 2021;38(10):4647–4654. 10.1093/molbev/msab199 34320186 PMC8476166

[ref-38] Mohr KH : 88. Familie: Chrysomelidae.In: Freude, H., Harde, K. W. & Lohse, G. A. (eds.) *Die Käfer Mitteleuropas.*Band 9. Goecke & Evers, Krefeld,1966;95–280.

[ref-17] Oatley G Denton A Howard C : Sanger Tree of Life HMW DNA extraction: automated MagAttract v.2. *protocols.io.* 2023a. 10.17504/protocols.io.kxygx3y4dg8j/v1

[ref-18] Oatley G Sampaio F Howard C : Sanger Tree of Life fragmented DNA clean up: automated SPRI. *protocols.io.* 2023b. 10.17504/protocols.io.q26g7p1wkgwz/v1

[ref-19] Rao SSP Huntley MH Durand NC : A 3D map of the human genome at kilobase resolution reveals principles of chromatin looping. *Cell.* 2014;159(7):1665–1680. 10.1016/j.cell.2014.11.021 25497547 PMC5635824

[ref-39] Rheinheimer J Hassler M : Die Blattkäfer Baden-Württembergs.Karlsruhe: Kleinsteuber Books,2018;928. Reference Source

[ref-20] Rhie A McCarthy SA Fedrigo O : Towards complete and error-free genome assemblies of all vertebrate species. *Nature.* 2021;592(7856):737–746. 10.1038/s41586-021-03451-0 33911273 PMC8081667

[ref-21] Rhie A Walenz BP Koren S : Merqury: reference-free quality, completeness, and phasing assessment for genome assemblies. *Genome Biol.* 2020;21(1): 245. 10.1186/s13059-020-02134-9 32928274 PMC7488777

[ref-22] Simão FA Waterhouse RM Ioannidis P : BUSCO: assessing genome assembly and annotation completeness with single-copy orthologs. *Bioinformatics.* 2015;31(19):3210–3212. 10.1093/bioinformatics/btv351 26059717

[ref-41] Steinhausen WR : 116. Familie: Chrysomelidae.In: KIausnitzer, B. (ed.) *Die Larven der Käfer Mitteleuropas*. 2. Band. Goecke & Evers, Krefeld,1994;231–314.

[ref-23] Surana P Muffato M Qi G : Sanger-tol/readmapping: sanger-tol/readmapping v1.1.0 - hebridean black (1.1.0). *Zenodo.* 2023a. 10.5281/zenodo.7755669

[ref-24] Surana P Muffato M Sadasivan Baby C : sanger-tol/genomenote (v1.0.dev). *Zenodo.* 2023b. 10.5281/zenodo.6785935

[ref-25] Todorovic M Sampaio F Howard C : Sanger Tree of Life HMW DNA fragmentation: diagenode Megaruptor ^®^3 for PacBio HiFi. *protocols.io.* 2023. 10.17504/protocols.io.8epv5x2zjg1b/v1

[ref-26] Uliano-Silva M Ferreira JGRN Krasheninnikova K : MitoHiFi: a python pipeline for mitochondrial genome assembly from PacBio high fidelity reads. *BMC Bioinformatics.* 2023;24(1): 288. 10.1186/s12859-023-05385-y 37464285 PMC10354987

[ref-27] UniProt Consortium: UniProt: a worldwide hub of protein knowledge. * Nucleic Acids Res.* 2019;47(D1):D506–D515. 10.1093/nar/gky1049 30395287 PMC6323992

[ref-28] Vasimuddin M Misra S Li H : Efficient architecture-aware acceleration of BWA-MEM for multicore systems. In: *2019 IEEE International Parallel and Distributed Processing Symposium (IPDPS)*. IEEE,2019;314–324. 10.1109/IPDPS.2019.00041

[ref-42] Warchałowski A : The palaearctic chrysomelidae. identification keys.Natura Optima Dux Foundation, Warszawa,2010;1–2:1212. Reference Source

[ref-29] Wellcome Sanger Institute: The genome sequence of the willow leaf beetle, *Lochmaea capreae* Linnaeus, 1758. European Nucleotide Archive, [dataset], accession number PRJEB59073,2023.

[ref-30] Zhou C McCarthy SA Durbin R : YaHS: yet another Hi-C scaffolding tool. *Bioinformatics.* 2023;39(1): btac808. 10.1093/bioinformatics/btac808 36525368 PMC9848053

