# Supplementary material for: The genome sequence of the willow leaf beetle, Lochmaea capreae Linnaeus, 1758
Source: Wellcome Open Res. 2024 Oct 17;9:304. Originally published 2024 Jun 11. [Version 2] doi: 10.12688/wellcomeopenres.22424.2 (PMC11503005; doi:10.12688/wellcomeopenres.22424.2)
Supplement: Peer review history

# Reviewer report: Reviewer response for version 2

**DOI:** 10.21956/wellcomeopenres.25604.r106117

**Published:** 2024-10-28

**Authors:** Doga Cedded

**Affiliations:** 1 University of Göttingen, Göttingen, Germany

The data note by Barclay et al. reports a high-quality genome assembly from *Lochmaea capreae. *The background information is appropriate for the insect. The methodology for the genome assembly is sufficiently described and adequate for the study. The authors also provide a detailed quality assessment of the assembled genome, with a k-mer completeness of 100% and a BUSCO score of 99.0%. I do not have any further comments regarding the data report.

Are sufficient details of methods and materials provided to allow replication by others?

Yes

Is the rationale for creating the dataset(s) clearly described?

Yes

Are the datasets clearly presented in a useable and accessible format?

Yes

Are the protocols appropriate and is the work technically sound?

Yes

Reviewer Expertise:

I work on coleopteran pests using RNAi and RNA-seq methods.

I confirm that I have read this submission and believe that I have an appropriate level of expertise to confirm that it is of an acceptable scientific standard.

# Reviewer report: Reviewer response for version 2

**DOI:** 10.21956/wellcomeopenres.25604.r105763

**Published:** 2024-10-24

**Authors:** Lapo Ragionieri

**Affiliations:** 1 Competence Centre for Plant Health, Competence Centre for Plant Health, Bolzano, South Tyrol, Italy; 2 University of Cologne, Cologne, Germany

The paper is considerably improved and can be accepted in its current state.

Are sufficient details of methods and materials provided to allow replication by others?

Partly

Is the rationale for creating the dataset(s) clearly described?

Partly

Are the datasets clearly presented in a useable and accessible format?

Yes

Are the protocols appropriate and is the work technically sound?

Yes

Reviewer Expertise:

GENOMIC; TRANSCRIPTOMIC AND PROTEOMIC

I confirm that I have read this submission and believe that I have an appropriate level of expertise to confirm that it is of an acceptable scientific standard.

# Reviewer report: Reviewer response for version 2

**DOI:** 10.21956/wellcomeopenres.25604.r105764

**Published:** 2024-10-21

**Authors:** Manee M Manee

**Affiliations:** 1 King Abdulaziz City for Science and Technology, Riyadh, Saudi Arabia

I am happy with the current form of this manuscript.

Are sufficient details of methods and materials provided to allow replication by others?

Yes

Is the rationale for creating the dataset(s) clearly described?

Yes

Are the datasets clearly presented in a useable and accessible format?

Yes

Are the protocols appropriate and is the work technically sound?

Yes

Reviewer Expertise:

bioinformatics, genomics, and evolutionary biology

I confirm that I have read this submission and believe that I have an appropriate level of expertise to confirm that it is of an acceptable scientific standard.

# Reviewer report: Reviewer response for version 1

**DOI:** 10.21956/wellcomeopenres.24703.r90747

**Published:** 2024-08-08

**Authors:** Lapo Ragionieri

**Affiliations:** 1 Competence Centre for Plant Health, Competence Centre for Plant Health, Bolzano, South Tyrol, Italy; 2 University of Cologne, Cologne, Germany

The data note "The genome sequence of the willow leaf beetle, *Lochmaea capreae* Linnaeus, 1758" by Barclay and co-authors describes the sequencing and genome assembly of *Lochmaea capreae*. The authors utilized Pacific Biosciences HiFi circular consensus DNA sequencing to produce an initial assembly, which was subsequently scaffolded using Hi-C. This resulted in a genome assembly at the chromosome level, with nearly all scaffolds assigned to chromosomes. The genome annotation is also included, combined with RNAseq data.

 The genome assembly is notably robust, characterized by a high degree of completeness and accuracy. The presentation of the results is clear, but the aims should be more clearly explained, especially regarding why this species was selected. For instance, is this a potential pest species for Salicaceae plants? The genome of this species can provide information about genes related to detoxification, digestion, and plant defense counteraction. Therefore, are the results obtained in this study potentially useful for studying adaptation in this species?

 Moreover, I think the information obtained in this data note is potentially useful for studies based on comparative genomics, such as evolutionary relationships and genetic diversity among beetles, a group of insects with a limited number of studied species.

 Below are some more specific comments and suggestions:

**Keywords:** The keywords need to be different from the title otherwise are useless.

**Species identification: **the author should cite the literature used.

**Mitochondrial genome**: I strongly recommend submitting the genome separated and with an independent accession number. Moreover, the authors described the methods but did not provide any information about the mitochondrial genome annotation (coding genes, rRNAs, and tRNAs). I would expect that the authors submit the annotations obtained by MITOS separately.

**Annotation**: The authors should identify and quantify the transposable elements.

**Command lines**: It is common practice to include the command lines used with all software. I believe this information can be very useful for the readers.

Are sufficient details of methods and materials provided to allow replication by others?

Partly

Is the rationale for creating the dataset(s) clearly described?

Partly

Are the datasets clearly presented in a useable and accessible format?

Yes

Are the protocols appropriate and is the work technically sound?

Yes

Reviewer Expertise:

GENOMIC; TRANSCRIPTOMIC AND PROTEOMIC

I confirm that I have read this submission and believe that I have an appropriate level of expertise to confirm that it is of an acceptable scientific standard, however I have significant reservations, as outlined above.

# Reviewer report: Reviewer response for version 1

**DOI:** 10.21956/wellcomeopenres.24703.r89720

**Published:** 2024-08-06

**Authors:** Manee M Manee

**Affiliations:** 1 King Abdulaziz City for Science and Technology, Riyadh, Saudi Arabia

The genome sequence of the willow leaf beetle, *Lochmaea capreae*, was assembled from a female specimen collected at Bookham Commons, England. The genome is approximately 534.7 megabases in length, with most of the assembly scaffolded into 17 chromosomal pseudomolecules. The mitochondrial genome was also assembled, with a length of 18.85 kilobases. Gene annotation identified 12,254 protein-coding genes and 1,518 non-coding genes. The final assembly is of high quality, with a QV of 68.5 and a BUSCO completeness of 99.0%. I am satisfied with the manuscript in its current form.

Are sufficient details of methods and materials provided to allow replication by others?

Yes

Is the rationale for creating the dataset(s) clearly described?

Yes

Are the datasets clearly presented in a useable and accessible format?

Yes

Are the protocols appropriate and is the work technically sound?

Yes

Reviewer Expertise:

bioinformatics, genomics, and evolutionary biology

I confirm that I have read this submission and believe that I have an appropriate level of expertise to confirm that it is of an acceptable scientific standard.
